# Strict target blood pressure management for reducing the stroke risk according to 2017 ACC/AHA blood pressure guideline

**DOI:** 10.18632/aging.102207

**Published:** 2019-08-27

**Authors:** Peng Zhao, Jie Liu, Conglin Wang, Wei Zhao, Yanqiu Zhang, Hongfei Gu, Jun Tu, Jinghua Wang, Xianjia Ning

**Affiliations:** 1 Department of Neurology, Tianjin Medical University General Hospital, Tianjin 300052, China; 2 Department of Epidemiology, Tianjin Neurological Institute, Tianjin 300052, China; 3 Tianjin Neurological Institute, Key Laboratory of Post-Neuroinjury Neuro-repair and Regeneration in Central Nervous System, Ministry of Education and Tianjin City, Tianjin 300052, China; 4 Department of Geriatrics, Tianjin Medical University General Hospital, Tianjin 300052, China; 5 Department of Neurology, Tianjin TEDA Hospital, Tianjin 300457, China; 6 Department of Neurology, Tianjin Nankai Hospital, Tianjin 300100, China; 7 Department of Neurosurgery, Tianjin Haibin People’s Hospital, Tianjin 300280, China

**Keywords:** blood pressure, stroke, risk factors, cohort study, epidemiology

## Abstract

Background and Purpose: We explored the new BP thresholds and their impact on first-ever stroke risk determinations.

Results: During a mean following-up period of 21.85 years, 638 first-ever strokes occurred among 3906 participants. After adjusting for covariates, the hazard ratios for ischemic stroke (IS) in men aged <60 years were significant higher in participants with elevated BP, stage 1 hypertension, and stage 2 hypertension than normal BP (all P<0.05); an increased risk of intracerebral hemorrhage (ICH) was also observed for those with stage 2 hypertension. Similarly, in women aged, the risk of stroke increased for those with stage 2 hypertension both in <60 years and in ≥60 years. Moreover, more than 60% of incident strokes were attributed to systolic BP (SBP) ≥120mmHg and diastolic BP (DBP) <80mmHg in men aged <60 years.

Conclusions: Elevated BP increases the risk of developing stroke, particularly in the absence of routine BP measurements and hypertension treatment. A strict BP management target (SBP, <120 mmHg; DBP, <80 mmHg) should be adopted for young and middle-aged men.

Methods: This population-based cohort study was conducted between October 1991 and January 2018. The association of BP categories, defined by the 2017 ACC/AHA BP guideline, with first-ever stroke risk was assessed using Cox regression models.

## INTRODUCTION

A 2016 statistical report indicated that the global lifetime stroke risk, for individuals ≥25 years old, is approximately 25% for both men and women; China had the highest estimated risk, at 39.3% [1]. Globally, there is a huge burden associated with strokes; there are an estimated 10.3 million new strokes, annually, accounting for 113 million disability-adjusted life years (DALYs) [2]. The stroke burden is particularly high in low- and middle-income countries where deaths associated with strokes account for approximately 75% of global stroke deaths and for >80% of DALYs; further, these countries have experienced increases in stroke incidence [3–5]. Our previous studies revealed that the incidence of first-ever stroke has been dramatically increasing in China’s rural population, especially among men aged 35–64 years, with an annual increase of 12% [6, 7].

High blood pressure (BP) is common in China, with the latest report indicating that the current prevalence of hypertension (defined as systolic BP [SBP]≥140 mmHg, diastolic BP [DBP]≥90 mmHg, or self-reported antihypertensive medication use within the previous 2 weeks) is 44.7% among residents 35–75 years old [8] and 23.2% among adults aged ≥18 years [9]. Our previous study demonstrated that the prevalence of hypertension in a rural population in 2011 was 51.7% among adults aged 35–74 years [10]. However, hypertension treatment and control rates are <50% and 20%, respectively, across different studies in China [11–14]; similarly, our study determined the treatment and control rates to be 43.8% and 12%, respectively [10].

BP is a powerful determinant of risk for both ischemic stroke (IS) and intracerebral hemorrhage (ICH). The evidence-based 2017 American College of Cardiology (ACC)/American Heart Association (AHA) Guideline for the Prevention, Detection, Evaluation, and Management of High Blood Pressure in Adults recommends intensive BP control for primary and secondary stroke prevention [15]. The guideline further proposes a target BP of <130/80 mmHg. In a recent meta-analysis, strong evidence indicated that BP control to <150/90 mmHg reduced stroke risk (relative risk [RR], 0.74;95% confidence interval [CI], 0.65–0.84]) and low- to moderate-strength evidence indicated that lower targets (≤140/85 mmHg) were associated with significant decreases in stroke risk (RR, 0.79;95% CI, 0.59–0.99) [16]. Recently, another report indicated that the new guidelines for the diagnosis and management of hypertension will likely influence hypertension management, globally, but especially in countries already facing an enormous public health challenge (using the previous definition of hypertension as BP ≥140/90 mmHg) [17]. However, data regarding the BP control target for reducing the stroke burden in China are scarce. In this study, we explored the optimal BP control target, according to the 2017 guideline, to decrease the risk of stroke in a population in China.

## RESULTS

**Figure 1:**
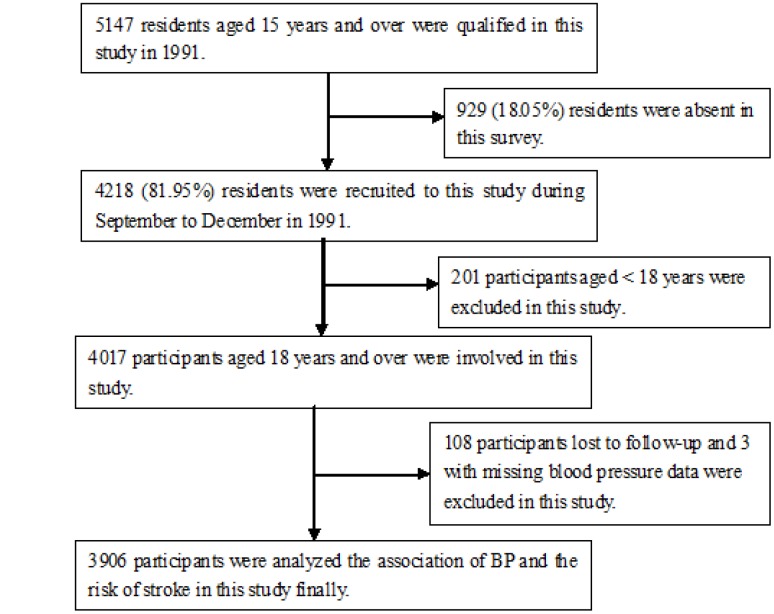
**Flow chart of participants.**

Overall, 5147 individuals were ≥15yearsold, and 4218 were recruited into this survey (response rate, 82%). Of these, 4017 individuals were ultimately enrolled, after excluding 201 individuals <18 years old. During the course of the study, 108 participants were lost to follow-up, and three with missing baseline BP data were removed from the BP analysis. Finally, a total of 3906 participants were evaluated to determine the association between BP and the incidence of first-ever stroke (Figure 1).

### Demographic features and baseline risk factors

During a mean following-up of 21.85 years (85,346 person-years), 3906 participants were involved in this cohort study; men accounted for 47% of the participants. The mean age of the participants was 41.7 years, overall; the average age of the men was 42.5 years and that of the women was 41.0 years. More than 60% of the individuals were <45 years old and had <7 years of education, only 3.5% of the individuals had a high school education. The mean SBP and DBP levels were 127.4 and 80 mmHg, respectively, overall; the corresponding levels were 127.9 and 80.6 mmHg in men and 126.9 and 79.4 mmHg in women. Using the 2017 hypertension guideline definitions, 21.4% of the participants had normal BPs and >70% participants had hypertension, at baseline. Moreover, the prevalence of overweight/obese individuals, current smoking, and current alcohol consumption was 25.9%, 25.7, and 15.4%, respectively (Table 1).

**Table 1:** Description of the demographical features at baseline among all participants by BP levels.

**Features**	**Total**	**Men**	**Women**	**P**
Participants	3906 (100)	1834 (47.0)	2072 (53.0)	—
Person-year	85346.51	38766.04	46580.47	—
Follow-up time, years	21.85	21.14	22.48	
Age, years	41.74 (16.58)	42.51 (16.89)	41.07 (16.28)	0.007
Age groups:				0.004
18~34 years	1582 (40.5)	720 (39.3)	862 (41.6)	
35~44 years	906 (23.2)	401 (21.9)	505 (24.4)	
45~54 years	466 (11.9)	238 (13.0)	228 (11.0)	
55~64 years	470 (12.0)	222 (12.1)	248 (12.0)	
65~74 years	320 (8.2)	168 (9.2)	152 (7.3)	
≥ 75 years	162 (4.1)	85 (4.6)	77 (3.7)	
Education attainment:				0.001
0 year	1588 (40.7)	675 (36.8)	913 (44.1)	
1~6 years	978 (25.0)	508 (27.7)	470 (22.7)	
7~9 years	1203 (30.8)	584 (31.8)	619 (29.9)	
≥ 10 years	137 (3.5)	67 (3.7)	70 (3.4)	
SBP, mmHg	127.39 (20.27)	127.92 (17.57)	126.92 (22.39)	0.120
DBP, mmHg	79.95 (11.41)	80.60 (10.59)	79.37 (12.06)	0.001
BP groups:				<0.001
Normal BP	835 (21.4)	291 (15.9)	544 (26.3)	
Elevated BP	314 (8.0)	146 (8.0)	168 (8.1)	
Stage 1 Hypertension	1559 (39.9)	820 (44.7)	739 (35.7)	
Stage 2 Hypertension	1198 (30.7)	577 (31.5)	621 (30.0)	
BMI, Kg/m^2^	22.59 (2.80)	22.32 (2.37)	22.83 (3.11)	<0.001
BMI groups:				<0.001
Normal	2897 (74.2)	1466 (79.9)	1431 (69.1)	
Over weight	847 (21.7)	332 (18.1)	515 (24.9)	
Obesity	162 (4.1)	36 (2.0)	126 (6.1)	
Smoking status:				<0.001
Never	2792 (71.5)	812 (44.3)	1980 (95.6)	
Ever	112 (2.9)	101 (5.5)	11 (0.5)	
Current	1002 (25.7)	921 (50.2)	81 (3.9)	
Drinking status:				<0.001
Never	3288 (84.2)	1241 (67.7)	2047 (98.8)	
Ever	16 (0.4)	16 (0.9)	0	
Current	602 (15.4)	577 (31.5)	25 (1.2)	

SD, standard deviation; SBP, systolic blood pressure; DBP, diastolic blood pressure; BMI, body mass index.

### Distribution of stroke risk factors, by BP group

Table 2 shows that the elevated BP levels were significantly associated with sex, age group, educational attainment, and BMI group (all, P<0.01). However, BP levels were not significantly associated with smoking or alcohol consumption.

**Table 2:** Distribution characteristics of stroke risk factors in this cohort study during 27-years following–up periods by BP groups.

**Characteristics**	**Normal BP**	**Elevated BP**	**Stage 1 hypertension**	**Stage 2 hypertension**	**P**
Gender, n (%):					<0.001
Men	291 (15.9)	146 (8.0)	820 (44.7)	577 (31.5)	
Women	544 (26.3)	168 (8.1)	739 (35.7)	621 (30.0)	
Total					
Age groups, n (%):					<0.001
< 60 years	785 (24.6)	286 (9.0)	1405 (44.1)	712 (22.3)	
≥ 60 years	50 (7.0)	28 (3.9)	154 (21.4)	486 (67.7)	
Education attainment, n (%):					<0.001
0 years	236 (14.9)	143 (9.0)	528 (33.2)	681 (42.9)	
1~6 years	239 (24.4)	82 (8.4)	385 (39.4)	272 (27.8)	
7~9 years	316 (26.3)	83 (6.9)	585 (48.6)	219 (18.2)	
> 9 years	44 (32.1)	6 (4.4)	61 (44.5)	26 (19.0)	
BMI groups, n (%):					<0.001
Normal	679 (23.4)	249 (8.6)	1199 (41.4)	770 (26.6)	
Over weight	137 (16.2)	54 (6.4)	316 (37.3)	340 (40.1)	
Obesity	19 (11.7)	11 (6.8)	44 (27.2)	88 (54.3)	
Smoking status, n (%):					0.089
Never	182 (18.2)	91 (9.1)	457 (45.6)	272 (27.1)	
Ever	7 (6.3)	3 (2.7)	43 (38.4)	59 (52.7)	
Current	646 (23.1)	220 (7.9)	1059 (37.9)	867 (31.1)	
Drinking status, n (%):					0.118
Never	105 (17.4)	47 (7.8)	276 (45.8)	174 (28.9)	
Ever	2 (12.5)	1 (6.3)	10 (62.5)	3 (18.8)	
Current	728 (22.1)	266 (8.1)	1273 (38.7)	1021 (31.1)	

### Incidence of first-ever stroke, based on demographic features, risk factors, and stroke type

Over the 27-year study period, 638 first-ever strokes were reported, including 404 ISs 121 ICHs, and 113 undetermined strokes. Thus, the overall incidence of first-ever stroke was 7.7/1000 person-years, including 9.7/1000 person-years for men and 5.8/1000 person-years for women; the incidence of IS was 4.5/1000 person-years and that of ICH was 1.4/1000 person-years. The incidences of normal BP (3.9/1000 person-years; 95% CI, 3.0–4.7), elevated BP (7.0/1000 person-years; 95% CI, 5.1–8.9), stage 1 hypertension (5.68/1000 person-years; 95% CI, 4.92–6.46), and stage 2 hypertension (15.53/1000 person-years; 95% CI, 13.85–17.21) were also determined. Moreover, the incidence of first-ever stroke, per 1000 person-years, increased with increasing BP and BMI levels, but decreased with increasing educational attainment (Table 3).

**Table 3:** Age-standardized incidence incidences of first-ever stroke in this cohort study during 27-years following–up periods by stroke subtypes (per 1000 person-year)^*^.

**Features**	**Stroke**	**Ischemic stroke**	**Hemorrhagic stroke**	**P**
Gender:				
Men	9.83 (5.87, 7.54)	5.95 (5.19, 6.73)	2.06 (1.61, 2.52)	<0.001
Women	5.57 (4.89, 6.24)	3.71 (3.16, 4.27)	0.88 (0.61, 1.15)	<0.001
Total	7.53 (6.90, 8.05)	4.73 (4.27, 5.19)	1.42 (1.17, 1.67)	<0.001
Age groups:				
< 60 years	5.35 (4.83, 5.87)	3.92 (3.48, 4.36)	1.11 (0.87, 1.34)	<0.001
≥ 60 years	26.64 (23.22, 30.06)	12.08 (9.77, 14.41)	4.23 (2.85, 5.60)	<0.001
Education attainment, years				
0	11.57 (10.24, 12.65)	6.46 (5.55, 7.38)	2.30 (1.76, 2.85)	<0.001
1~6	8.75 (7.52, 9.99)	5.83 (4.83, 6.84)	1.78 (1.22, 2.34)	<0.001
7~9	3.22 (2.59, 3.86)	2.63 (2.06, 3.21)	0.43 (0.20, 0.66)	<0.001
> 9	1.73 (0.35, 3.13)	1.45 (0.18, 2.72)	0.29 (-0.28, 0.86)	0.219
BP groups:				
Normal BP	2.87 (2.14, 3.60)	2.09 (1.51, 2.77)	0.53 (0.22, 0.85)	<0.001
Elevated BP	6.53 (4.64, 8.29)	4.85 (3.27, 6.43)	1.08 (0.33, 1.83)	<0.001
Stage 1 Hypertension	5.68 (4.92, 6.46)	3.69 (3.07, 4.31)	1.23 (0.87, 1.59)	<0.001
Stage 2 Hypertension	15.53 (13.85, 17.21)	9.09 (7.80, 10.38)	2.74 (2.03, 3.45)	<0.001
BMI groups:				
Normal	6.53 (5.90, 7.14)	4.11 (3.61, 4.61)	0.66 (0.86, 1.38)	<0.001
Over weight	9.86 (8.36, 11.17)	6.08 (4.97, 7.21)	2.42 (1.72, 3.13)	<0.001
Obesity	12.63 (8.77, 16.44)	9.23 (5.94, 12.51)	1.54 (0.19, 2.89)	<0.001
Smoking status:				
Never	6.57 (5.94, 7.23)	4.11 (3.60, 4.62)	1.21 (0.94, 1.49)	<0.001
Ever	16.33 (11.02, 21.60)	13.13 (8.39, 17.90)	2.27 (0.28, 4.25)	<0.001
Current	9.08 (7.81, 10.30)	5.61 (4.63, 6.60)	1.90 (1.33, 2.48)	<0.001
Drinking status:				
Never	7.24 (6.54, 7.78)	4.42 (3.93, 4.91)	1.41 (1.13, 1.68)	<0.001
Ever	27.64 (9.73, 45.49)	21.49 (5.65, 37.29)	6.14 (-2.39, 14.66)	0.177
Current	8.59 (7.07, 10.14)	5.95 (4.68, 7.23)	1.36 (0.75, 1.98)	<0.001

^*^presented as age-standardized incidence using WHO standardized population; 95%CI, 95% confidence interval; SBP, systolic blood pressure; DBP, diastolic blood pressure; BMI, body mass index.

### BP levels and incidence of first-ever stroke by sex, age, and stroke type

Table 4 shows that, relative to the normal BP group, the stroke risk increased significantly in the elevated BP group (increased 89%, P=0.001), the stage 1 hypertension group (increased 69%, P<0.001), and in the stage 2 hypertension group (increased 248%, P<0.001); the incidence of IS increased by 96% (P=0.003), 53% (P=0.014), and 225% (P<0.001), in the respective groups. Similar trends were found for the overall stroke risk for men in the elevated BP (HR, 2.08; 95% CI, 1.18–3.64; P=0.011), stage 1 hypertension(HR, 2.12; (5% CI,1.37–3.27; P=0.001), and stage 2 hypertension (HR, 4.36; 95% CI,2.82–6.75; P<0.001) groups. Among men, the trend also persisted for IS in those in the elevated BP (HR,2.14; 95% CI,1.10–4.16; P=0.025), stage 1 hypertension (HR, 1.87; 95% CI,1.11–3.16; P=0.019), and stage 2 hypertension (HR, 4.19; 95% CI,2.48–7.07; P<0.001) groups. However, among women, a significant association of BP levels with the risk of developing first-ever stroke was found only in the elevated BP and stage 2 hypertension groups, both for overall stroke and IS. Stage 2 hypertension increased the ICH risk in both men and women, increasing the stroke risk 2.8-fold in men and 1.6-fold in women.

**Table 4:** Adjusted hazard ratio of BP levels for the incidence of the first-ever stroke by sex and stroke types in this cohort study (95% CI).

**BP category**	**Stroke**	**IS**	**ICH**
Total:			
Normal BP	1.00	1.00	1.00
Elevated BP	1.89 (1.29, 2.78)^*^	1.96 (1.26, 3.04)^*^	1.58 (0.64, 3.94)
Stage 1 Hypertension	1.69 (1.27, 2.27)^*^	1.53 (1.09, 2.16)^*^	1.86 (0.96, 3.61)
Stage 2 Hypertension	3.48 (2.61, 4.64)^*^	3.25 (2.31, 4.57)^*^	3.16 (1.62, 6.16)^*^
Men:			
Normal BP	1.00	1.00	1.00
Elevated BP	2.08 (1.18, 3.64)^*^	2.14 (1.10, 4.16)^*^	1.97 (0.57, 6.82)
Stage 1 Hypertension	2.12 (1.37, 3.27)^*^	1.87 (1.11, 3.16)^*^	2.54 (0.99, 6.51)
Stage 2 Hypertension	4.36 (2.82, 6.75)^*^	4.19 (2.48, 7.07)^*^	3.81 (1.46, 9.96)^*^
Women:			
Normal BP	1.00	1.00	1.00
Elevated BP	1.89 (1.11, 3.20)^*^	2.00 (1.10, 3.64)^*^	1.25 (0.31, 5.05)
Stage 1 Hypertension	1.43 (0.95, 2.14)	1.39 (0.87, 2.22)	1.18 (0.43, 3.22)
Stage 2 Hypertension	2.91 (1.96, 4.32)^*^	2.70 (1.70, 4.29)^*^	2.60 (1.00, 6.78)^*^

Among men aged <60 years, the risk of developing first-ever stroke increased significantly in those with elevated BP, stage 1 hypertension, and stage 2 hypertension, for both stroke and IS. However, the risk of developing ICH increased significantly only for those with stage 2 hypertension (HR, 2.73; 95% CI,1.31–5.66; P=0.007). Similar findings were observed in men aged ≥60 years old. The risk of developing IS increased by 2.15-fold for those with elevated BP, 1.62-fold for those with stage 1 hypertension, and 4.4-fold for those with stage 2 hypertension (all P<0.05), respectively; the risk of developing ICH increased nearly 3-fold (P=0.014). The risk of developing the first-ever stroke in women <60 years old increased 1.5-fold for stroke and 1.7-fold for IS among those with stage 2 hypertension (P<0.001), but a similar correlation was not observed for ICH. Moreover, the IS risk among women aged ≥60 years only increased in those with stage 2 hypertension(HR, 2.98;95%CI, 1.08–8.26; P=0.035) (Table 5).

**Table 5:** Adjusted hazard ratio of BP levels for the incidence of the first-ever stroke by age, sex, and stroke types in this cohort study (95% CI).

**BP Category**	**Stroke**	**IS**	**ICH**
Total:			
< 60 years:			
Normal BP	1.00	1.00	1.00
Elevated BP	2.03 (1.34, 3.08)^*^	2.22 (1.37, 3.59)^*^	1.50 (0.57, 3.95)
Stage 1 Hypertension	1.70 (1.23, 2.35)^*^	1.71 (1.17, 2.50)^*^	1.73 (0.86, 3.51)
Stage 2 Hypertension	3.29 (2.36, 4.59)^*^	3.54 (2.40, 5.22)^*^	2.73 (1.31, 5.66)^*^
≥ 60 years			
Normal BP	1.00	1.00	1.00
Elevated BP	1.30 (0.48, 3.53)	1.11 (0.34, 3.63)	2.59 (0.16, 41.92)
Stage 1 Hypertension	1.36 (0.71, 2.63)	0.70 (0.32, 1.53)	2.66 (0.33, 21.21)
Stage 2 Hypertension	2.28 (1.24, 4.21)^*^	1.23 (0.61, 2.47)	3.74 (0.51, 27.78)
Men:			
< 60 years:			
Normal BP	1.00	1.00	1.00
Elevated BP	2.71 (1.45, 5.09)^*^	3.15 (1.47, 6.73)^*^	2.03 (0.50, 8.15)
Stage 1 Hypertension	2.49 (1.49, 4.16)^*^	2.62 (1.39, 4.96)^*^	2.79 (0.97, 8.01)
Stage 2 Hypertension	4.72 (2.79, 7.98)^*^	5.40 (2.83, 10.31)^*^	3.96 (1.33, 11.79)^*^
≥ 60 years:			
Normal BP	1.00	1.00	1.00
Elevated BP	0.62 (0.13, 3.02)	0.40 (0.05, 3.32)	2.01 (0.12, 32.67)
Stage 1 Hypertension	1.15 (0.50, 2.63)	0.45 (0.17, 1.22)	1.64 (0.20, 13.48)
Stage 2 Hypertension	2.06 (0.95, 4.47)	1.06 (0.45, 2.52)	2.16 (0.28, 16.42)
Women:			
< 60 years:			
Normal BP	1.00	1.00	1.00
Elevated BP	1.74 (0.98, 3.09)	1.87 (0.98, 3.58)	1.21 (0.30, 4.87)
Stage 1 Hypertension	1.25 (0.80, 1.96)	1.30 (0.78, 2.15)	0.93 (0.33, 2.66)
Stage 2 Hypertension	2.47 (1.57, 3.86)^*^	2.67 (1.60, 4.45)^*^	1.84 (0.65, 5.19)
≥ 60 years:			
Normal BP	1.00	1.00	1.00
Elevated BP	3.35 (0.82, 13.66)	3.00 (0.58, 15.35)	---
Stage 1 Hypertension	2.10 (0.70, 6.34)	1.58 (0.42, 5.92)	---
Stage 2 Hypertension	2.98 (1.08, 8.26)^*^	1.63 (0.49, 5.46)	---

**Figure 2:**
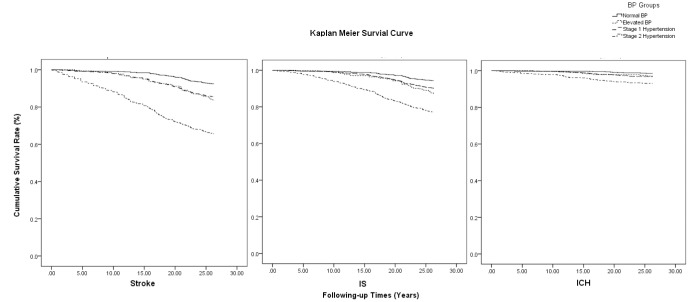
**Kaplan-Meier survival curve on association of BP levels with the risk of stroke by types.**

Additionally, elevated BP levels were associated with total stroke risk, as well as for IS and ICH risk, with respective log-rank values of 286.270,147.023, and 41.761 (all, P<0.001) (Figure 2).

### PAR% of abnormal BP on the incidence of first-ever stroke

**Figure 3:**
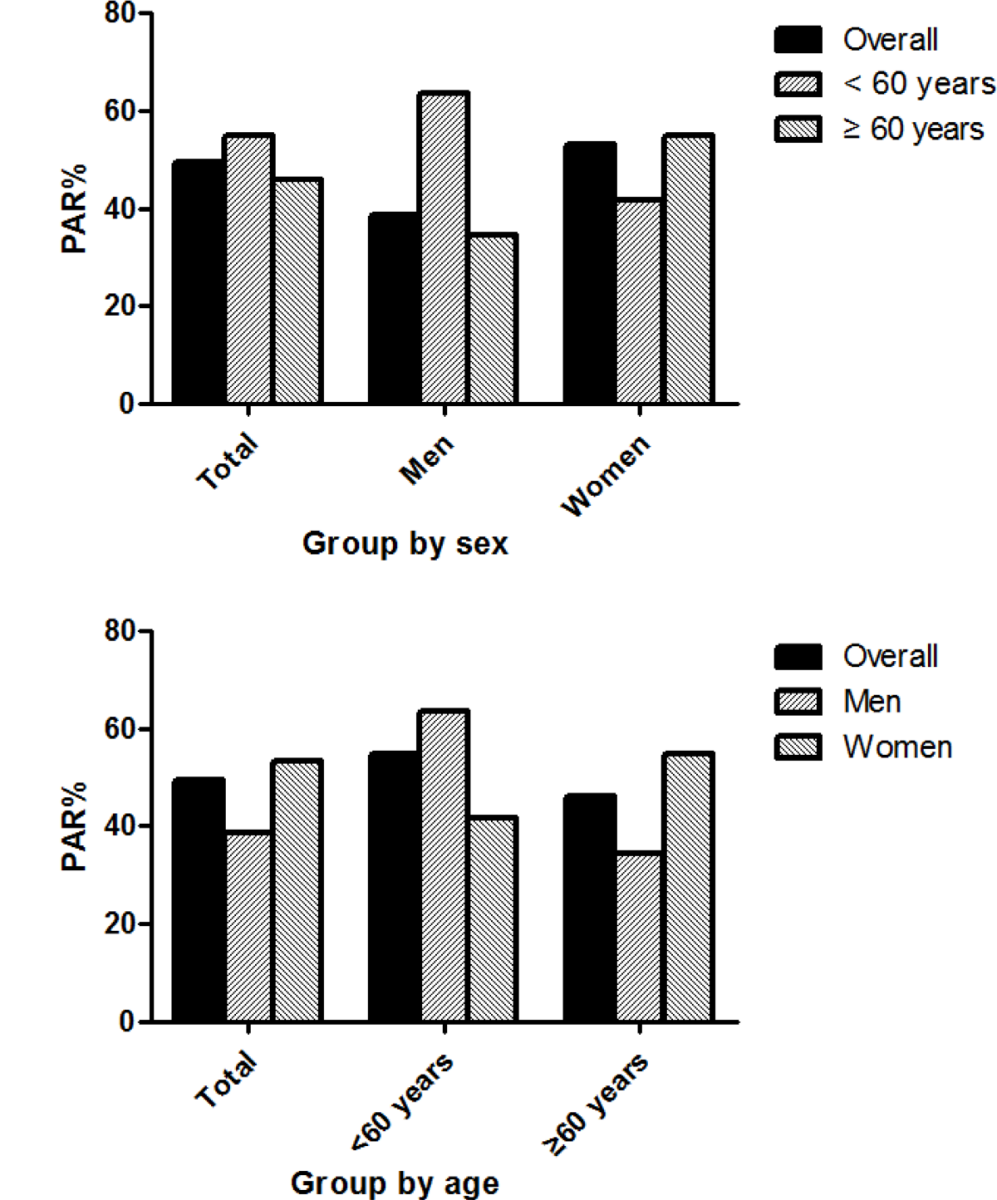
**Population attributable risk percent for the incidence of first-ever stroke associated with abnormal BP.**

Figure 3 shows that the PAR% for the incidence of first-ever stroke associated with abnormal BP (including elevated BP and stages 1 and 2 hypertension) was 49.4%, overall; the values for men and women were 38.5% and 53.3%, respectively. Among individuals <60 years old, the PAR was 54.8%, overall (63.6% for men and 41.7% for women). Among those aged ≥60 years, the corresponding PAR% values were 46.0% (overall risk), 34.4% (men), and 54.9% (women), respectively.

## DISCUSSION

In this 27-year prospective cohort study, we assessed the risk of developing first-ever stroke, based on the BP definitions in the 2017 ACC/AHA hypertension guideline. The findings indicate that elevated BP, stage 1 hypertension, and stage 2 hypertension were associated with significantly increased first-ever stroke risks, compared with normal BP. Additionally, there were sex, age, and stroke type distinctions regarding the impact of BP categories on developing stroke. Among men <60 years old, the risk of the developing IS increased significantly among those in all three abnormal BP classifications; however, only stage 2 hypertension appeared to significantly increase the risk of ICH. Among women <60 years old, there was an increased risk of IS observed among only those with stage 2 hypertension; further, there was no evidence of an increased risk of ICH associated with any BP category. There were no obvious associations between BP classifications and the risk of developing stroke among individuals aged ≥60 years old for either IS or ICH. The PAR% was higher in men than in women among individuals aged <60 years, but the opposite trend was found among those aged ≥60 years.

The definition of hypertension in the 2017 ACC/AHA guideline has been the subject of significant interest, globally. There has been concern regarding the increased number of patients defined as having hypertension based on this new definition that was reached, by consensus, among representatives from many countries [9, 17, 18–21]. In China, the prevalence of hypertension, based on the 2017 guideline, is twice as high as that based on the 2010 Chinese guideline; with the additional people captured under the new guideline, the prevalence of hypertension in China rose to 46.4%, whereas the control rate fell to 3.0% [9]. Additionally, the effects of the new BP classification on cardiovascular risk within a population is critical, especially in China.

Recently, several reports assessed CVD risk using the new BP stratification. The latest research from the American Coronary Artery Risk Development in Young Adults study indicated that, among young adults, those developing elevated blood pressure, stage 1 hypertension, or stage 2 hypertension (as defined by the blood pressure classification in the 2017 ACC/AHA guideline) before reaching 40 years of age had a significantly higher risk of subsequent CVD events than those with normal BP at 40 years of age. Thus, the new BP classification system may help identify young adults at a higher risk of CVD events [22]. Additionally, Korean young adults, aged 20–39 years with stage 1 or 2 hypertension had higher CVD risks than those with normal BP. Both men and women with elevated baseline BP, stage 1, or stage 2 hypertension, as defined by the new criteria, had higher risks of stroke than those with normal BP [23].

Liu and colleagues demonstrated that the effect of the 2017 ACC/AHA stage 1 hypertension definition on cardiovascular risk was evidenced in young and middle-aged Chinese adults, but not in those ≥60 years old. Among 35–59-year-old participants, the HR for CVD incidence when comparing individuals with stage 1 hypertension to those with SBPs <120 mmHg and DBPs <80 mmHg was 1.78 (95% CI, 1.50–2.11); that for coronary heart disease was 1.77 (95% CI, 1.33–2.36); that for stroke incidence was 1.79 (95% CI, 1.45–2.22); and that for CVD mortality was 2.50 (95% CI, 1.66–3.77) [24]. Similar to their findings, we found an association between the newly defined BP categories and first-ever stroke risk in young and middle-aged men and women in a rural Chinese population. Thus, we propose stricter BP control targets (SBP <120 mmHg and DBP <80 mmHg) for reducing the burden of stroke among young and middle-aged men in such rural populations. In these rural populations, there is a high prevalence of hypertension and a high stroke incidence, combined with lower rates of hypertension awareness, treatment, and control [10]. Adopting this BP control target is expected to reduce the incidence of first-ever stroke by 68% in men <60 years old.

In China, stroke carries the nation’s highest disease burden and was the nation’s leading cause of death in 2010, with 1.7 million deaths [25]. Our previous studies revealed that the incidence of first-ever stroke has been dramatically increasing among rural populations in China, especially among men aged 35–64 years (12% annual increase) [6, 7]. Moreover, the mean age of stroke onset in men has been decreasing by 0.28 years, annually, including annual declines of 0.56 years for ICH and 0.22 years for IS during the period between1992 and 2014 [26]. Further, hypertension is an established risk factor for stroke, and controlling BP is the most effective approach for preventing strokes [27–29]. Nevertheless, poor hypertension awareness, treatment, and control among young and middle-aged individuals have been reported in our prior studies [10]. Thus, executing strict BP target management (SBP <120mmHg and DBP <80mmHg) in these populations is critical for decreasing the incidence of first-ever stroke and reducing the disease burden of stroke in China.

The updated recommendations for defining and treating hypertension received a mixed response from several clinical societies [18–21, 30]. In part, this is because adopting the new guideline will result in a substantial increase in the reported prevalence of hypertension, in both the US and China. This would be accompanied by a marked increase in the number of individuals recommended to begin hypertension treatment and treatment would need to be intensified for several million patients [30]. However, a recent systematic review and meta-analysis indicated that lowering BP is associated with reduced risks of death and CVD if the baseline SBP is ≥140mmHg; at lower BP levels, treatment is not associated with any primary prevention benefit but might offer additional protection in patients with coronary heart disease [31]. We reported the BP levels just obtained at baseline in 1991, not a dynamic BP values. These may limited the popularization of these findings to board population. However, given that lower rates of hypertension awareness, treatment, and control, and poor health consciousness in this rural population, even though the dynamic BP values were absent, the stricter BP management target should be remained.

This study has several limitations. First, the study population was from a township in northern China, which is not representative of the overall national population. Further, the results may not necessarily be generalizable to other racial or ethnic populations. However, the prospective study design and lengthy study period may have reduced the impact of the study’s limited generalization. Second, the 85,000 person-years in the follow-up period did not fulfill the criteria of at least 100,000 person-years of observations recommended for population studies [32]. Third, we only assessed the risk of stroke using BP levels at baseline, without dynamic BP measurements. However, the low rates of hypertension awareness, treatment, and control, as well as the overall lack of health consciousness in this rural population, suggest that the strict BP management target should still be used, despite the lack of reporting of dynamic BP values. Finally, we did not collect detailed information regarding fasting glucose and lipids levels, dietary habits, or medication use; therefore, other possible determinants of stroke could not be assessed in this study.

## MATERIALS AND METHODS

### Study population and sampling method

This analysis was based on a 27-year population-based cohort study that began in1991 and was conducted in Tianjin, China; the study design was previously described [10]. Briefly, the study involved individuals participating in the Tianjin Brain Study, a population-based stroke surveillance study. The participants resided in 18 administrative villages in Tianjin, and 95% were low-income farmers. The primary source of income was grain production, with the residents having an annual per capita income of <100 USD in 1991 and <1000 USD in 2010 [33].

The sampling method used in this cohort study was also previously reported [34]. Briefly, the villages were grouped according to their geographic location (east, south, and north) and two villages from each geographic location were randomly sampled, using a stratified cluster sampling method. In these six villages, all adults aged ≥18years and without histories of cardiovascular disease (CVD) or stroke were recruited into the study.

The study protocol was approved by the ethics committee of Tianjin Medical University General Hospital (TMUGH); written informed consent was obtained from each participant.

### Baseline information

Individual demographic characteristics (including sex, age, and educational attainment), disease history (including hypertension, diabetes, stroke, and CVD), and lifestyle factors (including smoking and alcohol consumption status and physical activity), at baseline, were collected in 1991. All information was collected by local, trained research staff who conducted face-to-face interviews; the interviews also included physical examinations to determine BPs, heights, and body weights.

### Risk factor measurement and categorization

In this study, we assessed the association of stroke risk with baseline BP levels. BP was measured as previously described [10]. Briefly, standardized BP measurements were performed using a calibrated mercury sphygmomanometer, with the cuff size adjusted to the individual’s arm circumference. The cuff was placed on the arm at the level of the heart, and the BP was recorded as the mean of two measurements, 5 min apart, with the participant resting in the supine position. Each patient avoided caffeine, exercise, and smoking for at least 30 min prior to the measurements. The SBP and DBP values were determined according to Korotkoff sounds I and V. If the difference between the two consecutive readings was not within 10 mmHg (SBP) and/or 5mmHg (DBP), or if the measurement reached the criteria for hypertension, two further readings were obtained after the participant rested for an additional 20 min.

To evaluate stroke risk associated with the BP categories, individuals were stratified, according to the new guideline: normal BP (SBP,<120 mmHg; DBP,<80 mmHg), elevated BP (SBP, 120–129 mmHg; DBP,<80 mmHg), stage 1 hypertension (SBP, 130–139 mmHg; DBP, 80–89 mmHg), and stage 2 hypertension (SBP, ≥140mmHg or DBP, ≥90 mmHg). Diabetes, stroke, and CVD determinations were based on self-reported disease histories. Body mass indexes (BMIs) were calculated and used to categorize individuals as being normal weight (BMI <24 kg/m^2^), overweight (BMI = 24–27.9 kg/m^2^), or obese (BMI ≥28 kg/m^2^) [34].

### Stroke diagnosis and types

Stroke was defined as an acute-onset, focal neurological deficit of vascular etiology persisting for >24 h, including both ischemic and hemorrhagic subtypes [35]. Hemorrhagic stroke was defined as an ICH;IS was defined as a thrombotic brain infarction, cardioembolic stroke, or lacunar infarct; an undetermined stroke was defined as a stroke that could not be classified into either subtype. All strokes were symptomatic, with significant clinical symptoms and signs. Transient ischemic attacks and silent strokes (diagnosed by imaging, only) were excluded, but stroke patients with histories of transient ischemic attacks prior to a defined stroke were included. Patients demonstrating transient symptoms and having concurrent neuroimaging evidence of brain infarctions were considered as stroke cases, based on the “tissue” definition [36]. In the early phase of this study (1992–1998), the events were confirmed primarily based on clinical examinations by senior neurologists for non-hospitalized patients and using medical records for hospitalized patients.

### Stroke reporting

Stroke events were reported using thepredefined procedures reported previously [6]. Briefly, local, licensed village physicians reported initial stroke events to the community hospital within 24 h of onset. Within 72 h, community hospital physicians visited the surviving patients’ homes to confirm stroke events and obtain clinical feature information. Confirmed stroke events (imaging diagnosis) were reported monthly to TMUGH, and suspected events (no imaging performed) were reported in a timely manner. Finally, a TMUGH neurologist identified suspected cases through door-to-door interviews, as soon as possible.

### Statistical analysis

Continuous variables (age, SBP, DBP, and BMI) are presented as means and standard deviations (SDs); categorical variables are presented as frequencies with 95% CIs. Subgroup analyses were conducted to evaluate the first-ever stroke risk by age group (18–34 years, 35–44 years, 45–54 years, 55–64 years, 65–74 years, and ≥75 years), education level (illiterate [no formal education], 1–6 years, 7–9 years, and ≥10 years of formal education), BMI group (normal, overweight, and obese), smoking status (never smoked, previous smoker, and current smoker), and drinking status (never consumed alcohol, previously consumed alcohol, and currently consumes alcohol). Adjusted hazard ratios (HRs) for the incidence of overall stroke and each type, by BP category, were estimated using Cox proportional hazards models adjusted for age, sex, education level, BMI, smoking status, and drinking status. Moreover, a subgroup analysis was performed to detect the association of BP levels with stroke risk, by age (simplified to <60 years and ≥60 years). Diabetes was not analyzed in this study because there were too few patients with diabetes (n = 4), at baseline. Population attributable risk percent (PAR%) was used to estimate the proportion of incident stroke events attributable to abnormal BPs (including elevated BP, stage 1 hypertension, and stage 2 hypertension), which was calculated using the formula: PAR%=(I_t_-I_0_)/I_t_, where I_t_ denotes the total stroke incidence in the whole population during the study period; I_0_ denotes the stroke incidence among those with normal BP. All statistical analyses were performed using SPSS for Windows (version 15.0; SPSS, Chicago, IL, USA); a P-value < 0.05 was considered statistically significant.

## SUMMARY

To the best of our knowledge, this is the first prospective study aimed at quantitatively determining the first-ever stroke risk associated with the new hypertension guideline. Elevated BP can increase the risk of developing stroke if people do not undergo routine BP measurements and treat any detected hypertension. The BP management target for reducing the risk of incident IS and ICH in young and middle-aged men in China is an SBP <120mmHg and a DBP <80mmHg. However, for young and middle-aged women, we recommend a BP management target of SBP <140mmHg and DBP <90mmHg to decrease the incident IS risk. Therefore, an urgent focus on the early adoption of lifestyle interventions among young and middle-aged adults, especially for men, is needed to decrease the incidence of stroke in China. In addition, the recommended BP control target for reducing the burden of stroke among young and middle-aged men provides a valuable reference for other developing countries experiencing the serious disease burdens associated with chronic, non-infectious diseases, such as hypertension and stroke.
